# Targeted lapatinib anti-HER2/ErbB2 therapy resistance in breast cancer: opportunities to overcome a difficult problem

**DOI:** 10.20517/cdr.2019.92

**Published:** 2020-02-28

**Authors:** Reema Wahdan-Alaswad, Bolin Liu, Ann D. Thor

**Affiliations:** ^1^Department of Pathology, University of Colorado Anschutz Medical Campus, Aurora CO 80014, USA.; ^2^Department of Genetics, Stanley S. Scott Cancer Center, School of Medicine, Louisiana State University Health Sciences Center, New Orleans, LA 70112, USA.

**Keywords:** HER2/ErbB2, receptor tyrosine kinase, tyrosine kinase resistance, nuclear receptor, androgen receptor, lipid metabolism, programmed cell death-1 ligand, CDK 4/6 inhibitor

## Abstract

Approximately 20% of invasive breast cancers have upregulation/gene amplification of the oncogene human epidermal growth factor receptor-2 (HER2/ErbB2). Of these, some also express steroid receptors (the so-called Luminal B subtype), whereas others do not (the HER2 subtype). HER2 abnormal breast cancers are associated with a worse prognosis, chemotherapy resistance, and sensitivity to selected anti-HER2 targeted therapeutics. Transcriptional data from over 3000 invasive breast cancers suggest that this approach is overly simplistic; rather, the upregulation of HER2 expression resulting from gene amplification is a driver event that causes major transcriptional changes involving numerous genes and pathways in breast cancer cells. Most notably, this includes a shift from estrogenic dependence to regulatory controls driven by other nuclear receptors, particularly the androgen receptor. We discuss members of the HER receptor tyrosine kinase family, heterodimer formation, and downstream signaling, with a focus on HER2 associated pathology in breast carcinogenesis. The development and application of anti-HER2 drugs, including selected clinical trials, are discussed. In light of the many excellent reviews in the clinical literature, our emphasis is on recently developed and successful strategies to overcome targeted therapy resistance. These include combining anti-HER2 agents with programmed cell death-1 ligand or cyclin-dependent kinase 4/6 inhibitors, targeting crosstalk between HER2 and other nuclear receptors, lipid/cholesterol synthesis to inhibit receptor tyrosine kinase activation, and metformin, a broadly inhibitory drug. We seek to facilitate a better understanding of new approaches to overcome anti-HER2 drug resistance and encourage exploration of two other therapeutic interventions that may be clinically useful for HER+ invasive breast cancer patients.

## Introduction

### Human epidermal growth factor-2 positive breast cancer

Primary invasive breast cancers are characterized on the basis of their clinical, histopathologic, and marker data. This includes disease stage, histologic type, histologic grade, tumor size, and the expression or lack thereof of two steroid receptors [estrogen (ER) and progesterone (PgR)] and the human epidermal growth factor receptor-2 (HER2/ErbB2). HER2 overexpression/amplification occurs in 20%-30% of invasive breast cancer^[1]^. As a prognostic tumor marker, HER2 positivity (+) is independently associated with a worse outcome^[1]^. As a predictive marker, it guides treatment selection to optimize patient outcome^[2]^. The transmembrane receptor HER2 has no ligand; activation of its enzymatic tyrosine kinase activity is achieved by dimerization with other ligand-bound HER family receptors. HER2 positivity is associated with phenotypic and biologic aggression, characterized by an increase in cellular proliferation, motility, invasiveness, metastasis, angiogenesis, decreased apoptosis, and chemotherapy resistance as compared to luminal A (ER+/PgR+, HER2-) breast cancer^[3,4]^.

### HER2 receptor dimerization and signaling in breast cancer

The HER family proteins are type I transmembrane receptor tyrosine kinases (RTKs). Members include the epidermal growth factor receptor (EGFR/HER1/ErbB1), HER2 (ErbB2), HER3 (ErbB3), and HER4 (ErbB4). Each of the HER family receptors plays critical roles in the activation of intracellular signaling pathways in response to extracellular signaling, although, in the case of HER2, this is indirect via dimerization with other ligand bound receptors^[5]^. Ligands can display receptor specificity. For example, transforming growth factor-α, androgen receptor (AR), EGF, and Epigen bind to EGFR/HER1 or bind to one or more related receptors. Neuregulins 1-4 bind to HER3 and HER4. Heparin binding epidermal growth factor (HB-EGF), epiregulin, and β-cellulin bind to and activate EGFR/HER4. HER2 dimerization with alternate ligand-bound HER family receptors facilitates activation of its tyrosine kinase domain and intracellular signaling pathways, including PI3K/Akt/mTOR, Ras/Raf/MEK/ERK/MAPK, Src, and STAT. These critical signaling cascades regulate cell proliferation, migration, differentiation, cell motility, and apoptosis^[2,5-12]^.

## Classification of invasive breast cancer as HER2 positive, negative, or indeterminant: a predicate for targeted therapeutics

A robust and reliable determination of hormone receptor and HER2 “status” in newly diagnosed breast cancer is more difficult to achieve than it may seem. Some of this is due to clinical and surgical nuances of individual patient tumors, as well as the distance and time required for transportation of the biopsy, examination, and processing by pathology. Clinical teams should adhere to the most recent widely accepted practice guidelines, developed by a working group and codified by the College of American Pathologists and the American Society of Clinical Oncology (www.asco.org/breast-cancer-guidelines)^[13]^. These recommendations include the use of FDA approved reagents, test, and reporting methodology. Testing must be performed in a clinical laboratory improvement amendments-certified laboratory, using appropriate controls and validated assays. Each of these assays requires a pathologist to perform a semi-quantitative analysis of the cancer sample using evidence-based interpretive guidelines and visual microscopy^[14]^.

Despite national and international efforts to standardize reagents, protocols, and other variables, some variability among tissues, tests, labs, and pathologists is inevitable. For immunohistochemistry (IHC), positivity is determined by the visualization and pathologist ascribed quantitation of intense circumferential membranous positivity of invasive breast cancer cells, exceeding the FDA approved threshold for positivity. The application of digital microscopy and computer algorithms to semi-quantitatively score IHC stained samples of invasive breast cancer can also be used. This interpretive tool does not modify pre-analytic or post-analytic errors associated with other IHC methods. Digital scoring also introduces additional sources of error and the test still requires the selection invasive breast cancer cells by a pathologist for interpretation. Guidelines for in situ hybridization (ISH) testing show a preference for dual probe assays. These generate data reflecting a ratio of the number of intra-nuclear HER2 to centromeric Ch17 signals in a sufficient number of invasive breast cancer cells. ISH assay interpretation can be difficult, as it is not always easy to be assured that each cell counted is representative of an invasive breast cancer focus. Thus, ISH testing shares some of the analytic error issues with IHC.

For HER2 testing, each methodology and reagent utilizes FDA approved cut-points for positive (also called 3+ for IHC), indeterminate (also called 2+ for IHC), and negative (0 or 1+ by IHC). In cases that score indeterminate by either method, testing using the alternative method is required. Positivity by either method is considered evidence of HER2 alteration and qualifies the patient for anti-HER2 targeted therapy. Some laboratories perform two methods of HER2 testing on all cases [even negative (0) and positive (3+)], to reduce error^[15,16]^.

IHC and fluorescence in situ hybridization data reproducibility, cancer phenotype, and biology, as well as responsivity to chemotherapy, hormone therapy, and/or targeted anti-HER2 therapy, are strongly influenced by HER2 heterogeneity in cell sub-clones within the primary cancer, among primary, regional, or distant metastases, and over time in a single patient in response to treatment associated cell selection^[17]^. Thus, retesting for HER2 status is typically recommended with cancer recurrence or metastases, if targeted therapeutics are considered.

In addition to defining a response to anti-HER2 targeted therapeutics, overexpression of HER2 by IHC and/or amplification of the HER2 gene has also been shown to predict a more favorable response to anthracycline-based chemotherapy and resistance to other forms of chemotherapy^[18,19]^.

Amplification/overexpression of HER2 (HER2 positive) likely reflects genomic evolution within a breast cancer. HER2+, pretreated, and metastatic breast cancers (MBCs) frequently show alterations of additional targetable genomic defects in comparison to the primary neoplasm. These may act as driver mutations and provide therapeutic opportunities for intervention^[20]^. Yates *et al*.^[20]^ make the case for performing gene sequencing or other assays to detect targetable mutations in HER2+ breast cancer cases, as a more “personalized medicine approach” for individual patients. Sequencing data can also separate recurrent disease from a secondary breast malignancy.

## HER2 specific targeted therapeutics

**Figure 1:**
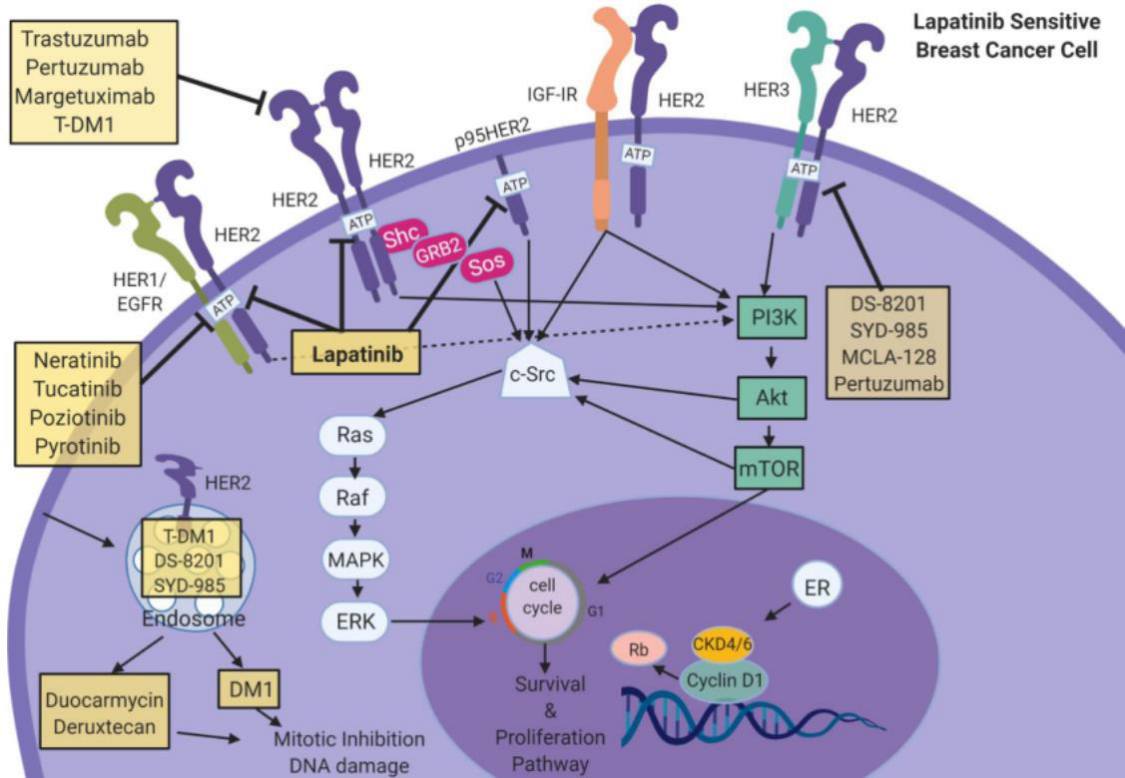
Signaling cross-talk within HER family receptors. HER family RTKs or growth factor receptors (EGFR (HER1/ErbB1), HER3 (ErbB3), HER4 (ErbB4), and IGF-IR) are able to activate several oncogenic signaling pathways such as Ras/Raf/MAPK/ERK or PI3K/Akt/mTOR to stimulate growth and proliferation. Direct inhibitors can inhibit HER2 including trastuzumab, pertuzumab, margetuzumab, or T-DM1 to block proliferation and cell growth. TDM-1, DS-8201, and SYD-985 are internalized through caveolae-mediated endocytosis to inhibit mitosis and facilitate DNA damage. Antibody conjugates that target HER3:HER2 complex include DS-8201, SYD-985, MCLA-128 which enhance cell mediated cytotoxicity in the cell. Direct antibody conjugates against EGFR neratinib, tucatinib, poziotinib, pyrotinib work to attenuate proliferation. Lapatinib, a HER2/EGFR inhibitor, attenuates cell proliferation, cell cycle regulation, and down-stream pathways. Images were created using Biorender.com. CDK 4/6: Cyclin-dependent kinase4/6; DM1: Derivative of Maytansine 1; EGFR: epidermal growth factor receptor; ER: estrogen receptor; HER2: Human epidermal growth factor receptor 2; HER3: Human epidermal growth factor receptor 3; IGF-1R: insulin-like growth factor 1 receptor; mTOR: mammalian Target of Rapamycin; PI3K: Phosphoinositide 3-kinase; Rb: Retinoblastoma protein. ERK: extracellular-signal regulated kinase

Targeted anti-HER2 drugs have been used in HER2+ breast cancer patients for several decades. Both early and late stage disease patients have benefitted from this approach, which is widely reviewed elsewhere^[11,16,21-24]^. Anti-HER2 drugs include trastuzumab, pertuzumab, margetuximab, trastuzumab trastuzumab emtansine DM1 (T-DM1), and lapatinib. Trastuzumab is a humanized monoclonal antibody (MoAb) that directly binds to the extracellular domain of HER2 to block receptor activation and downstream signaling [Figure 1]^[11,21,22,25-27]^. Trastuzumab has revolutionized the treatment of HER2+ breast cancer, representing one of the most remarkable examples of targeted therapy in oncology. However, issues of the best chemotherapy companion with trastuzumab, cardiac toxicities, and clinical resistance still require tremendous efforts by researchers^[28]^.

Pertuzumab is a humanized MoAb that binds to the extracellular domain II of HER2, preventing homo- or heterodimerization with itself or other members of HER family, especially HER3^[29,30]^. Pertuzumab was granted approval for use in combination with trastuzumab and chemotherapy as adjuvant treatments for HER2+ breast cancer. While reports show a favorable disease-free survival (DFS) and overall survival (OS) for pertuzumab with trastuzumab and chemotherapy in MBC, these reports warrant further studies.

Neratinib is an irreversible tyrosine kinase inhibitor of EGFR, HER1, HER2, and HER4. The FDA has approved the use of neratinib for extended adjuvant therapy for early stage HER2+ breast cancer following adjuvant trastuzumab-based therapy. While neratinib significantly improves treatment outcomes and has manageable toxicity in stage I-III HER2+ breast cancer patients, additional studies regarding neratinib-resistance remain yet to be understood^[31]^.

Tucatinib is an oral highly selective inhibitor of HER2 tyrosine kinase with minimal inhibition of EGFR^[32]^. In a Phase Ib dose-escalation trial, tucatinib in combination with trastuzumab and capecitabine showed encouraging anti-tumor activity in patients with HER2+ MBC^[33]^. The addition of tucatinib to trastuzumab and capecitabine resulted in better DFS and OS than placebo^[33]^. Adverse effects of toxicity were noted when higher levels of tucatinib were used.

Margetuximab is a monoclonal anti-HER2 antibody against the same epitope as trastuzumab but the Fc domain of the antibody is optimized for a better antibody-dependant conjugation/binding^[34]^. In a Phase III study, patients treated with margetuximab + chemotherapy demonstrated significant disease free progression [5.8 months *vs*. 4.9 months, hazard ratio (HR) = 0.76; 95%CI: 0.59-0.98, *P* = 0.033]^[34]^. Margetuximab was superior to trastuzumab on DFS in HER2-positive MBC. Margetuximab was able to enhance innate immunity *in vitro* and antibody-dependent cytotoxicity^[35,36]^. Margetuximab activates both innate and adaptive immunity, creating coordinated engagement of HER2-targeted immunity^[34]^.

Trastuzumab-DM1 (T-DM1, ado-trastuzumab emtansine) is an antibody-drug conjugate of trastuzumab covalently linked to a maytansine derivate (DM1). It binds to HER2 on cancer cell membranes, primarily targeting chemotherapy delivery to the malignant cells^[29,37,38]^. It is generally used in patients who have already received trastuzumab as well as chemotherapy (paclitaxel or docetaxel), as it reduces toxicity to healthy cells.

The antibody-drug conjugate Fam-trastuzumab deruxtecan-nxki (DS-8201, Trastuzumab deruxtecan) targets HER2^[39]^. Fam-trastuzumab deruxtecan-nxki (DS-8201) is now approved by the FDA and showed promising anti-tumor activity in patients with HER2+ MBC. This agent outperformed trastuzumab emtansine; however, there were reports that indicate a rise in resistance to trastuzumab emtansine based on inadequate binding of the antibody to HER2 receptor^[40]^. While DS-8201 provides a high level of clinical activity in patients with HER2+ MBC, this agent was associated with a substantial risk of interstitial lung disease.

Lapatinib is a selective, reversible, ATP-competitive tyrosine kinase inhibitor that binds to both HER2 and the epidermal growth factor (EGFR/HER1)^[21,27,29,41-51]^. Lapatinib is often combined with other agents, including chemotherapy (capecitabine, gemcitabine, or vinorelbine), anti-HER2 targeting agents (trastuzumab), and endocrine therapy (e.g., letrozole) for high risk metastatic HER2-positive patients who demonstrate clinical progression (resistance) to trastuzumab^[48]^. It is also preferentially administered to patients with brain metastasis, which occurs in approximately half of breast cancer patients with HER2+ disease.

### Primary mechanisms of resistance to anti-HER2 therapies

Many potential mechanisms to anti-HER2 therapy have been described that lead to the reactivation of the HER2 pathway and its downstream signaling. Resistance to trastuzumab and combinations with various chemotherapies have been described^[52]^. These mechanisms of resistance to anti-HER2 therapy can be primary, where there is a lack of positive response to the therapy (intrinsic resistance) or as disease progression after the initial clinical benefit (secondary/acquired resistance). The presence of primary or acquired resistance to anti-HER2 treatments remains a significant challenge for HER2 positive breast cancer patients. Nearly all patients with metastatic HER2-positive breast cancer eventually progress on anti-HER2 therapy due to *de novo* or acquired resistance. For example, primary resistance to single-agent trastuzumab accounts for 66%-88% of HER2-overexpressing breast cancer and can occur in the neoadjuvant as well as the adjuvant setting^[53]^. Many potential resistance mechanisms to anti-HER2 therapy ultimately result in the reactivation of the HER2 pathway or its downstream signaling, through pathway redundancy or simultaneous activation of alternate pathways^[54]^. Mechanisms of resistance to anti-HER2 therapies include: (1) incomplete blockade of the HER2 receptor or overexpression of HER2 ligands to activate compensatory mechanisms within HER family (such as HER3)^[55]^; (2) activation of other tyrosine kinase receptors (RTKs)/growth factor signaling pathways, such as the insulin-like growth factor 1^[56]^, tyrosine-protein kinase MET (MET)^[57]^, or PI3K/Akt/mTOR^[58,59]^; (3) alterations of the binding site, secondary to masking epitopes or secretion of truncated HER2 receptors (P95)^[60]^; (4) loss of down-stream controllers (e.g., phosphatase and tensin homolog)^[61]^; and (5) activation of alternate factors including stem cells, chemokine receptors, metabolites, microenvironmental factors, and host-related factors (reviewed in Ref.^[10]^). While the majority of resistance to first-line anti-HER2 therapy (such as trastuzumab) is primary, a more thorough understanding of HER2 therapy resistance (primary or acquired) is necessary to develop new strategies to improve patient outcomes. The ability to recognize primary resistance soon after the initiation of therapy enables earlier use of alternative therapeutic strategies to overcome resistance (as reviewed in Ref.^[52]^). Given the plethora of primary resistance literature, our report focuses on secondary resistance and reviews new treatment paradigms that combine optimized HER2-targeted therapies to improve outcomes for HER2-positive invasive breast cancer patients.

### Lapatinib combination therapy for HER2+ breast cancer

Despite success with the introduction of anti-HER2 as a dual inhibitor, single agent in the treatment of advanced breast cancer, most patients ultimately develop progressive (lapatinib-resistant) MBC and succumb to the disease. Recommendations for the use of HER2-targeted therapies in HER2+ breast cancer^[23,43,45,52,62]^ are presented in Table 1. Additional information is available at: www.asco.org/breast-cancer-guidelines. A growing body of evidence also indicates that primary and acquired resistance to lapatinib therapy is frequent and of clinical consequence. Resistance is typically associated with shifts in compensatory signaling pathways, the acquisition of new driver mutations, tumor heterogeneity, and changes in the tumor microenvironment or innate host immunity (reviewed in Ref.^[23,43,52]^).

**Table 1:** Characteristics of key Phase II/III clinical trials with lapatinib and trastuzumab in HER2+ breast cancers

Agent	Clinical trial	Phase	Treatment comparison	Sample size	Clinical result pCR	Reference
Lapatinib	NeoALTO	III	Paclitaxel + T	465	24.70%	[22]
			Paclitaxel + L		29.50%	[44]
			Paclitaxel + T + L		51.30%	
	ALTO	III	Standard Chemo + T	8381	NR	[128]
			Standard Chemo + L			[129]
			Standard Chemo + T --> L			[130]
			Standard Chemo + T + L			
	CALGB 40601	II	Paclitaxel + T	305	46%	[131]
			Paclitaxel + T + L		56%	
			Paclitaxel + L		32%	
	Lapatinib TEACH	II	Standard Chemo--> L	3147	NR	[132]
			Standard Chemo-->observation			
	CHERLOB	II	Standard Chemo + T	121	26.30%	[48]
			Standard Chemo + L		25.00%	
			Standard Chemo + L + T		46.70%	
	NSABP B-41 Trial	III	Standard Chemo + L	519	53.20%	[133]
			Standard Chemo + T		52.50%	
			Standard Chemo + L + T		62.00%	
	TRIO US B-07	II	Standard Chemo + T	92	57.00%	[134]
			Standard Chemo + L + T		67.00%	
	The GeparQuinto Trial	III	Standard Chemo + L	615	22.70%	[135]
			Standard Chemo + T		30.30%	

Standard Chemo: may include epirubicin, cyclophosphamide, 5-FU, Adriamycin, methotrexate, docetaxel, or carboplatin; T: trastuzumab; L: lapatinib; NR: not reported, ongoing clinical result

## Mechanisms of lapatinib resistance

**Figure 2:**
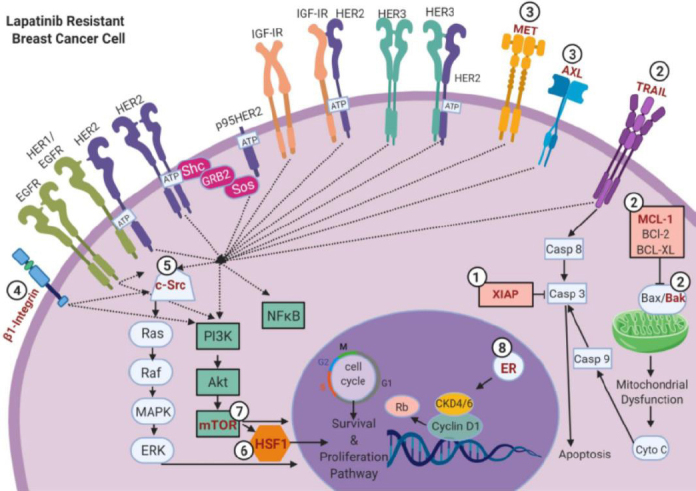
Mechanism of resistance to lapatinib. Myriad potential mechanisms of resistance to lapatinib have been reported. (1) Alterations in cell death cascade through up-regulation of XIAP, which blocks caspase 3-mediated apoptosis. (2) MCL-2 hyperactivation or TRAIL receptor can further attenuate apoptosis functions. (3) Activation of compensatory pathways may be mediated by up-regulation of RTKs, such as HER3, through the formation of HER2/HER3 heterodimerization; MET, the phosphorylation of which is stimulated by HGF; AXL overexpression; and IGF-IR dimerization. These receptors can dimerize with HER2 to further promote proliferative cues. (4) Activation of β1-integris can further promote activation of Src or PI3K/Akt/mTOR signaling axis to enhance cell proliferation. (5) Components of the PI3K/Akt/mTOR pathway, Src family kinases, and PTK6 may dampen pro-apoptotic function and enhance cell proliferation. (6 and 7) HSF1 and mTOR can mediate cancer cell survival and metastasis. (8) Modulation of ER increase cell proliferation. Images were created using Biorender.com. Casp 3: caspase 3; Casp 8: caspase 8; Casp 9: caspase 9; CDK 4/6: cyclin-dependent kinase4/6; Cyto C: cytochrome C; EGFR: epidermal growth factor receptor; ER: estrogen receptor; HER2: human epidermal growth factor receptor 2; HER3: human epidermal growth factor receptor 3; IGF-1R: insulin-like growth factor 1 receptor; MCF-1: myeloid leukemia 1; mTOR: mammalian target of rapamycin; PI3K: phosphoinositide 3-kinase; Rb: retinoblastoma protein; TRAIL: tumor necrosis factor-related apoptosis-inducing ligand, XIAP: X inhibitor of apoptosis protein; RTKs: receptor tyrosine kinases; HGF: hepatocyte growth factor; PTK6: protein tyrosine kinase 6; MAPK: mitogen activated protein kinase; MCL: mantle cell lymphoma; MET: tyrosine-protein kinase MET; AXL: AXL receptor tyrosine kinase; IGF-IR: insulin-like growth factor receptor 1; BCL-XL: b-cell lymphoma extra-large; HSF1: Heat shock factor 1; NFκB: nuclear factor kappa-beta; ERK: extracellular signal-regulated kinase

Lapatinib has shown remarkable efficacy in the treatment of trastuzumab-refractory HER2+ breast cancer, at least for the short term. However, a significant proportion of these patients will develop progressive disease due to acquired or *de novo* resistance [Figure 2]. Newer agents that include neratinib, tucatinib, margetuximab, and antibody-drug conjugate trastuzumab deruxtecan (DS-8201) are currently being evaluated in clinical trials to decipher whether these agents can benefit HER2+ breast cancer patients who are resistant to trastuzumab or lapatinib-resistance.

A frequent mechanism of lapatinib resistance is through inhibition of apoptotic response pathways and pro-growth signaling, through hyperactivation or overexpression of genes including X inhibitor of apoptosis protein (XIAP)^[63]^, myeloid leukemia 1^[64]^, and the tumor necrosis factor-related apoptosis-inducing ligand^[45,50,63,64]^. Overexpression with activation of other tyrosine receptor kinases [such as Axl, MET, insulin-like growth factor receptor 1 (IGF-1R), and vascular endothelial growth factor (VEGF)] is another common mechanism of resistance. Targeting of these other RTK receptors with tyrosine kinase inhibitors, along with lapatinib, often restores sensitivity^[49,65-67]^. Activation of pro-proliferative signaling pathways such as PI3K/Akt/mTOR, Src, or MAPK is also associated with a worse prognosis and resistance in HER2+ breast cancer patients. Combinatorial treatment with agents that target these pathways, such as everolimus, AZD0530, or dasatinib improve survival in lapatinib- and trastuzumab-refractory patients^[68-70]^. The development of marker or gene assays, to better predict treatment response tailored to specific genetic and genomic features of the breast cancer would be helpful to prevent anti-HER2 treatment resistance. Genetic profiling to identify targetable or driver mutations in recurrent cancer or MBC is also increasingly being used in higher stage or refractory patients to better define opportunities for intervention with a personalized approach.

## New strategies for overcoming lapatinib resistance

**Figure 3:**
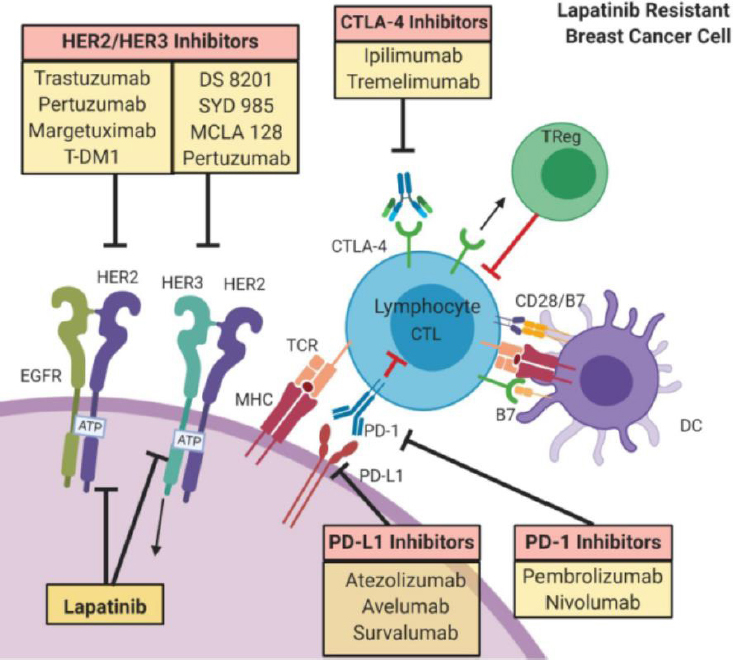
Immune regulation in HER2+ breast cancer. Immunomodulation of lapatinib-resistant HER2+ breast cancer cells act through the expression of PD-L1 by cancer cells which inhibits the cytotoxic activity of CD8+ lymphocytes. Different compounds acting on HER2- or HER2-signaling partners for clinically or preclinical agents are shown in yellow boxes. Main inhibitors target: (1) HER2 or HER2/HER3 and HER2/EGFR receptors; (2) CTLA-4 inhibitors that target CTLA-4 on lymphocytes; (3) PD-L1 inhibitors that target PD-L1 on cancer cell; or (4) PD-1 inhibitors that target PD-1 on lymphocyte. Images were created using Biorender.com. HER2: human epidermal growth factor receptor 2; HER3: human epidermal growth factor receptor 3; CTLA-4: cytotoxic T-lymphocyte antigen 4; CTL: cytotoxic T-lymphocyte; DC: dendritic cell; MHC: major histocompatibility complex; PD-1: programmed cell death-1; PD-L1: programmed cell death-1 ligand; TCR: T cell receptor; Tregs: regulatory T cells; EGFR: epidermal growth factor receptor; T-DM1:trastuzumab emtansine DM1

Maturation in the fields of immuno-oncology and breast cancer research has drastically changed our understanding of the immune microenvironment and surveillance mechanisms that facilitate disease progression [Figure 3]^[71]^. Early data suggest patterns of immunogenicity are quite heterogeneous patient to patient, within a single cancer mass, and among primary, recurrent, and metastatic disease. In addition, alterations of immune competence induced by socioeconomic, environmental, and nutritional characteristics; chronic diseases; and chemotherapy or other anti-cancer agents may also inhibit the ability of a single patient to immunologically “attack” their breast cancer. Preclinical and clinical studies centered on immunotherapy have particularly focused on triple negative and HER2+ breast cancers, given their worse prognosis and fewer treatment options. We highlight some recent advances below (see also excellent reviews elsewhere^[72]^).

Invasive breast cancer with tumor-infiltrating lymphocytes (TILs), as compared to similar tumors without an immunologic response, have been associated with a more favorable outcome to anti-HER2 therapy. Immuno-phenotyping has shown that the lymphocytic population of cells are diverse and include natural killer (NK) cells, macrophages, dendritic cells (DCs), adaptive immune cells, and CD8+ cytotoxic T lymphocytes (CTLs). These cells often surround and permeate the infiltrative tumor mass, most prominently surrounding individual tumor cells or cell clusters. NK cells are part of the innate immune systems, responding quickly to perceived threats. They possess the unique ability to kill cancer cells without priming or prior activation, in contrast to CTLs. NK cells also secrete cytokines that act on macrophages and DCs to enhance the anti-cancer immune response. NK cells touch and respond to inhibitory or activating receptors on the membrane of cancer cells, and will respond robustly against breast cancer cells that lose their major histocompatibility complex class I protein (MHC1) receptors. CTLs play a critical role in the host’s immune repertoire necessary for an effective anti-cancer response.

Cancer cells also demonstrate a plethora of immune escape mechanisms including: alterations or downregulation of the major histocompatibility complex MHC1 protein, the human leukocyte antigen class I protein, and the T cell receptor (TCR). To counter these immune-suppressive changes, immunotherapeutic interventions have sought to enhance recognition signaling and immune processing induced by the presence of “foreign” cancerous cells; to upregulate or activate the immune system; and to block or reduce the immunosuppressive CTL activity. Strategies against breast cancer include: (1) targeting PD-L1 (programmed cell death receptor ligand 1), an immune checkpoint inhibitor; (2) inactivating the programmed cell death protein 1 (PD-1), which downregulates the immune system and promotes self-tolerance by CTL; and (3) inhibiting the cytotoxic T lymphocyte antigen-4 (CTLA-4) [Figure 3]. There are currently two FDA approved drugs available, one against PD-L1 (atezolizumab)^[29]^ and the other targeting PD-1 (pembrolizumab)^[73]^. Each can be used against invasive breast cancer, and are most frequently administered to patients with triple-negative or HER2+ disease.

Many of these agents are used in otherwise treatment resistant breast cancer patients, with several showing promise in clinical trials. For example, the Pembrolizumab plus trastuzumab in trastuzumab-resistant, advanced, HER2-positive breast cancer (PANACEA) trial evaluated trastuzumab and pembrolizumab as a single arm in trastuzumab-resistant, advanced HER2+ invasive breast cancer patients. A response was observed in 15% of PD-L1 positive patients^[52,74]^. This trial has led to optimism about active immunotherapy using a similar immune checkpoint blockade for HER2+ breast cancer resistant to lapatinib therapy, or in less heavily pretreated PD-L1 patients. TILs act as powerful predictive and prognostic biomarkers in neoadjuvant-treated HER2+ breast cancer, more so in the trastazumab-treated subgroup over lapatinib-treated cohort^[40]^. Interactions of host immunity with HER2-targeted agents, such as margetuximab, were reported in HER2+ breast cancer^[75]^. Because of the ability of margetuximab to enhance t-cell response and antibody response, the use of this agent was found to be superior to trastuzumab and may be beneficial in the lapatinib-resistant setting^[52]^. Directly targeting PD-L1 or PD-1 has also been an effective combinatorial strategy in some breast cancer patients^[74,76]^. In HER2+ breast cancer patients, the goal of immunotherapy would be to upregulate the immune response and overcome resistance to anti-HER2 based treatment regimens.

### Cyclin-dependent kinase 4/6 inhibitors and lapatinib demonstrate efficacy against ER+/HER2+ MBC

**Figure 4:**
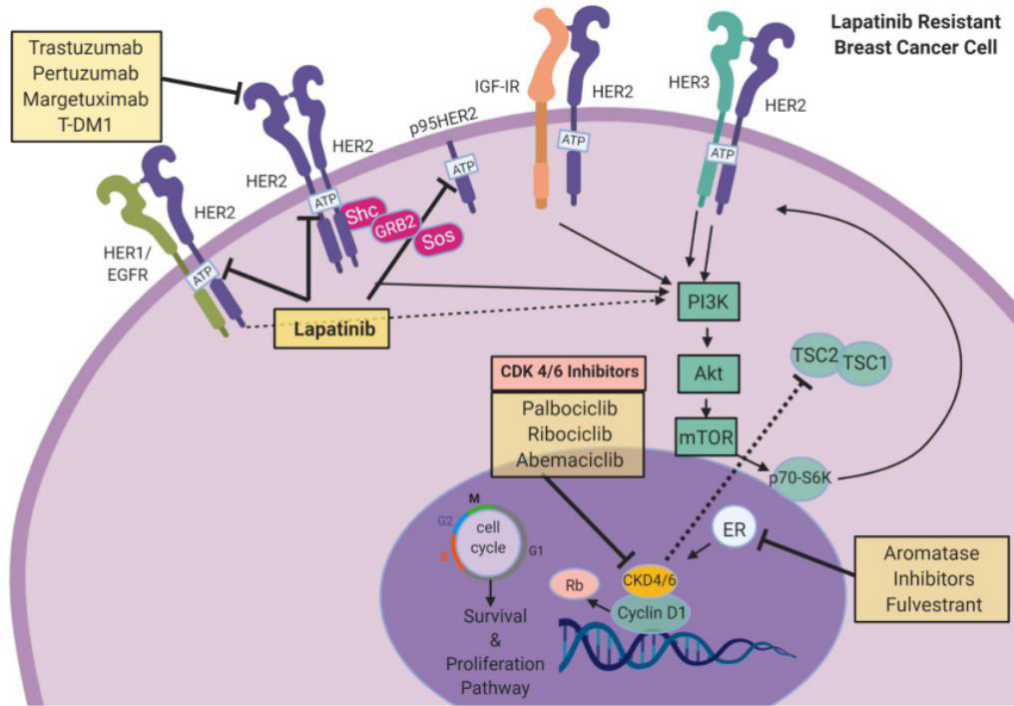
Targeting CDK 4/6 in HER2+ breast cancer. CDK4 and CDK6 play critical roles in the cell cycle. HER2 signaling or ER signaling will trigger an increase in cyclin D1 levels, which will activate CDK 4/6. Activation of the CDK 4/6 complex includes cyclin E in late G1 phase. Cyclin D1 and CDK 4/6 also phosphorylate and inactivate Rb protein, which in turn enables cell cycle progression and increase in cellular proliferation. Use of CDK 4/6 inhibitors palbociclib, ribociclib, and abemaciclib attenuates HER2-mediated induction of proliferation. Use of PI3K/Akt/mTOR inhibitors synergistically reduces cell division. Combining CDK 4/6 inhibitors with ER inhibitors or AIs can further inhibit breast cancer cell growth. Images were created using Biorender.com. CDK 4/6: cyclin-dependent kinase 4/6; EGFR: epidermal growth factor receptor; ER: estrogen receptor; HER2: human epidermal growth factor receptor 2; HER3: human epidermal growth factor receptor 3; IGF-1R: insulin-like growth factor 1 receptor; mTOR: mammalian target of rapamycin; PI3K: phosphoinositide 3-kinase; Rb: retinoblastoma protein; TSC 1/2: TSC complex subunit 1/2; AIs: aromatase inhibitors; T-DM1: trastuzumab emtansine DM1.

Cell cycle dysfunction is a characteristic of cancer. Most notably, cancer cells lose regulatory controls that induce cell cycle arrest or slow down cell division, particularly in the presence of genetic damage, resulting in a hyper-proliferative phenotype and cancer growth. The tumor doubling time (Td) for breast cancer can be especially rapid with recurrence or metastasis, in the absence of other therapy, with nearly half showing a Td of < 25 days, one third growing at a moderate rate (Td of 26-75 days), and 15% growing slowly, Td of > 76 days^[77]^. Based on the characteristic of rapid growth, agents targeting the replicative machinery have proven useful in breast cancer patients. Many of these are directed against enzymes known as cyclin-dependent kinases (CDKs), which modulate cell cycle progression and thus the ability of a cancer cell to divide^[52,78-81]^. For example, the tumor suppressor protein retinoblastoma (Rb) controls cell cycle progression from G1 to S. The CDK 4/6-retinoblastoma axis is especially important in HER2+ breast cancers that express ER. In these patients, direct inhibitors of CDK 4/6 have shown benefit and may be synergistic with other drugs, including tamoxifen or trastuzumab^[78,79,82,83]^. Palbociclib, ribociclib, and abemaciclib are highly selective reversible inhibitors of CDK 4/6 that have been approved by the FDA for use in HER2+ ER+ MBC patients [Figure 4]. New data suggest that CDK 4/6 inhibitors may also be useful in HER2+, ER/PgR- cases as well. For example, in late stage trastuzumab-resistant breast cancer patients, the addition of CDK 4/6 inhibitors to trastuzumab was associated with enhanced treatment response^[79]^. Combination regimens including CDK 4/6 inhibitors in earlier stage HER2+ disease are being used in the PATINA and MONARCHER trials^[79,82,83]^. Of these, abemaciclib has shown potent synergistic effects in preclinical studies when combined with lapatinib^[84]^. Other clinical trials that evaluate CDK 4/6 inhibitors in combination with anti-HER2 agents include NCT02530424, NCT02947685, NCT02448420, NCT02675231, NCT02657343, NCT03054363, and NCT03054363. The use of CDK 4/6 inhibitors in patients with lapatinib-resistance has not been widely tested. Methodologies to identify or quantify targets for CDK 4/6 inhibitors on cancer cells, as well as other biomarkers assays to evaluate weaknesses of immune competence in the breast cancer microenvironment, would be especially useful as the field matures.

### Co-targeting nuclear receptors and the HER2 pathway

The nuclear receptors (NRs) ER and PgR play critical roles in steroid hormone signaling, as well as the development and progression of both NR+ and/or HER2+ breast cancer^[85]^. HER2 cross-talk with NR is frequent and bidirectional. It may result in resistance to agents targeting either receptor class if given as a single agent. The majority of HER2+ breast cancer tumors express steroid receptors, and, for those patients, targeting of ER and HER2 with combinatorial protocols has been a useful strategy^[86]^. Others believe that HER2 is “not a cancer subtype, but rather a pan-cancer event” with upregulation of androgenic signaling and a unique transcriptional pattern^[16]^. While AR and anti-AR drugs are important and relatively new areas of breast cancer research^[87-90]^, the vitamin D3 receptor^[91,92]^, glucocorticoid receptor^[93,94]^, retinoic acid receptor alpha^[95,96]^, and other NR are less well explored in the pathogenesis of this disease.

**Figure 5:**
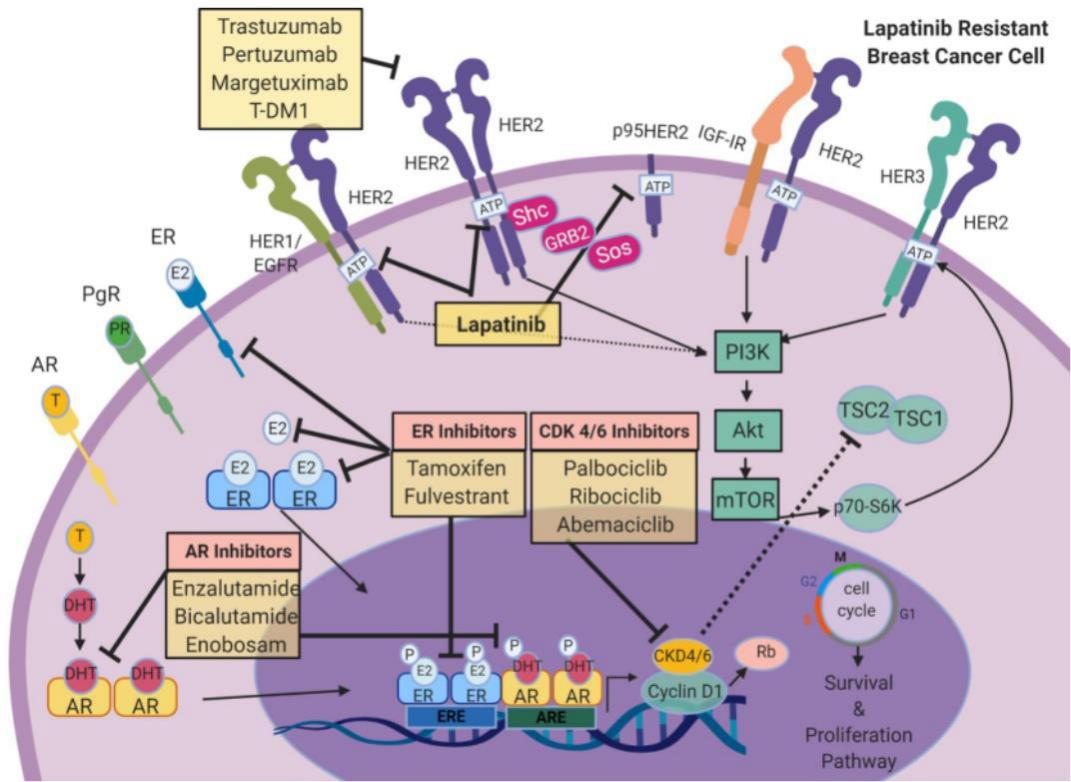
Bi-directional cross-talk with HER2 and nuclear receptors. While ER signaling is the dominant driver of cell proliferation and survival in breast cancer, alternate receptors such as PgR and AR can also influence HER2-mediated cell proliferation in breast cancer. Direct inhibitors against ER (tamoxifen or fulvestrant) or AR (enzalutamide, bicalutamide, or enobosarm) can work in concert with lapatinib to directly target HER2 signaling and attenuate cell cycle regulation. Combining lapatinib and nuclear receptor inhibitors with CDK4/6 inhibitors (palbociclib, ribociclib, or abemaciclib) can synergistically attenuate HER2-mediated cell growth. Images were created using Biorender.com. AR: androgen receptor; CDK 4/6: cyclin-dependent kinase 4/6; DHT: dihydrotestosterone; EGFR: epidermal growth factor receptor; E2: estradiol/estrogen; ER: estrogen receptor; HER2: human epidermal growth factor receptor 2; HER3: human epidermal growth factor receptor 3; IGF-1R: insulin-like growth factor 1 receptor; mTOR: mammalian target of rapamycin; PI3K: phosphoinositide 3-kinase; PgR: progesterone; Rb: retinoblastoma protein; T: testosterone; TSC 1/2: TSC complex subunit 1/2; ERE: estrogen response element; ARE: androgen response element; T-DM1: trastuzumab emtansine DM1; PR: progesterone receptor

AR signaling is associated with sensitivity to lapatinib^[97]^ [Figure 5]. During the development of lapatinib-resistance, reactivation of ER signaling and dependence, with downregulation of androgenic signaling and dependence has been observed. ER activation is associated with a specific and predictable pattern of gene expression including upregulation of IGF-IR, cyclin D1, insulin-like growth factor II, and VEGF^[84,98]^. These data provide a rationale to target downstream intermediates such as cyclin D1 by CDK 4/6 inhibitors in ER+/HER2+ breast tumors^[78,79,82-84]^. The American Society of Clinical Oncology currently recommends a combination of lapatinib and chemotherapy (such as capecitabine), along with hormone therapy in advanced HR+/HER2+ breast cancer^[13,48]^. While concomitant treatment of anti-HER2 agents with fulvestrant can directly attenuate pro-proliferative cues by estrogen-signaling, targeting of the androgen receptor is also feasible with the development of new selective therapeutics.

Methodology and reagents to quantify AR expression by breast cancer cells are in development. The Richer lab demonstrated that AR is overexpressed/activated in 77% of HER2+ tumors^[89,90,99]^. They also reported that a high ratio of AR to ER expression better predicts tumor biology and treatment response, as compared to AR data alone. Preclinical studies have shown that an AR antagonist, enzalutamide, reduces breast cancer proliferation in HER2+, trastuzumab-resistant cell lines^[100,101]^. Phase II clinical trials have shown that enzalutamide in combination with trastuzumab has shown significant benefit for high stage or locally advanced HER2+/AR+ breast cancer (NCT02091960^[102]^, https://clinicaltrials.gov/ct2/show/NCT02091960). Other AR inhibitors (such as bicalutamide or enobosarm: Gtx-024) have also been combined with lapatinib to restore lapatinib-sensitivity in HER2+/HR+ breast cancer patients^[103]^. Results from these trials, as well as companion studies, will likely reveal which breast cancer patients would most benefit from this approach.

### Lipid reprogramming and lapatinib resistance

Lipids, and more specifically cholesterol, plays a critical role in carcinogenesis. Women with altered lipid metabolism, most notably hypercholesterolemia in the setting of obesity and often type II diabetes, have a significantly higher risk of breast cancer. Lowering of cholesterol levels, most often with statin drugs, has been shown to reduce this risk. For example, a longitudinal study of over 1 million women in Europe has shown women with drug induced lowering of cholesterol show a significant reduction in breast cancer incidence^[104]^.

Cholesterol-rich domains in the plasma membrane, known as lipid rafts, provide membranous tethering of receptors and other molecules that enhance signal transduction and intracellular transport^[105]^. The cholesterol rich ganglioside (GM1) lipid rafts are of particular importance in HER2+ or triple negative breast cancer cells, as they localize and stabilize transmembrane RTKs, facilitating ligand binding, activation, and downstream signaling^[106]^. Many of the HER2 RTK family members require GM1 lipid rafts for oncogenicity, including the EGFR and HER2^[107]^. Spatial deregulation and massive upregulation of these RTKs is common in breast cancer, further enhancing ligand binding, homo- and heterodimer formation, kinase activation, phosphorylation of the cytoplasmic domain, and regulation of downstream signaling.

**Figure 6:**
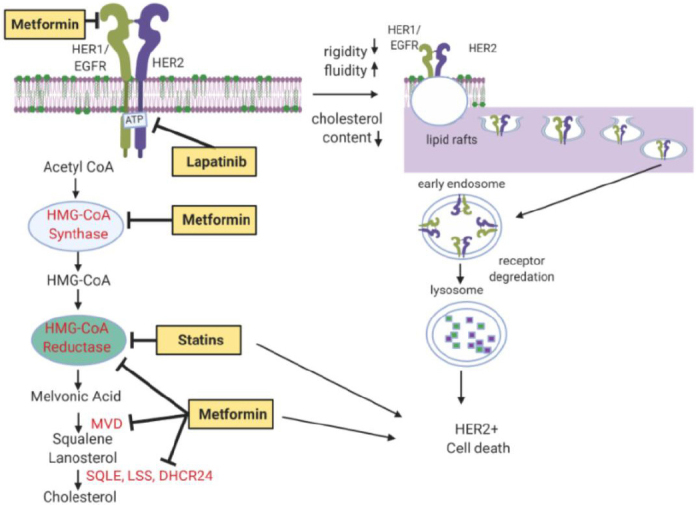
Disruption of HER2 signaling by destabilizing cholesterol content and lipid raft formation. Statins and anti-diabetic agent, metformin, can inhibit HMGCR and disrupt cholesterol signaling. Lapatinib directly targets HER2. Metformin can also target key enzymes critical to cholesterol synthesis pathways, which include HMGCoA, HMGCR, MVD, SQLE, LSS, and DHCR24 (red). Images were created using Biorender.com. EGFR: epidermal growth factor receptor; HER2: human epidermal growth factor receptor 2; HMGCoA: 3-hydroxy, 3-methylglutaryl-CoA; HMGCR: HMG-CoA reductase; MVD: mevalonate diphosphate decarboxylase; SQLE: squalene expoxidase; LSS: lanosterol synthase; DHCR24: 24-dehydrocholesterol reductase

Disruption of lipid rafts, via selective blockade of cholesterol biosynthesis through statin associated inhibition of HMGCo-A reductase^[108,109]^ or other agents to induce cholesterol biosynthesis downregulate, destabilize, and deactivate RTK, inhibiting breast cancer cell growth and inducing programmed cell death^[110-112]^. Inhibition of GM1 lipid rafts by methyl-beta-cyclodextrin (MβCD), a cholesterol chelator, has shown similar effects including apoptosis of breast cancer^[113]^. Due to toxicity, MβCD cannot be used in living organisms. We also showed that the relatively non-toxic oral anti-diabetic drug metformin targets numerous key enzymes, including β-Hydroxy β-methylglutaryl-CoA (HMG-CoA) reductase, in the cholesterol biosynthesis pathway^[114]^ and that it downregulates RTK activation and oncogenic signaling. We also demonstrated that metformin blocks synthesis of precursor fatty acids through upregulation of miR193b^[115]^. Metformin is synergistic with statins to downregulate lipid rafts and destabilize RTKs, downregulating pro-oncogenic signaling and inducing cell death in triple negative breast cancer [Figure 6]^[114,116,117]^. Other approaches have also been used to target and destabilize lipid rafts, for example drugs that induce translational modifications such as myristolation, disrupt cellular signaling, and disrupt protein-protein interactions^[118]^.

The aforementioned agents that promote dysregulation of cholesterol homeostasis have also demonstrated the ability to inhibit or kill HER2+ breast cancer cells^[119]^. The mevalonate pathway and squalene epoxidase, both overexpressed in HER2 breast tumors, are also attractive drug targets for lapatinib-resistant HER2+ breast cancer^[119,120]^. Simvastatin has shown both antiproliferative and pro-apoptotic effects against HER2+ breast cancer^[110]^. Lovastatin, in combination with lapatinib, also reduced HER2+ breast cancer cell growth as a xenograft model *in vivo*. These findings confirm that targeting cholesterol (and lipid rafts) may enhance spatial regulation of RTKs function and reduce pro-carcinogenic signaling in HER2+ breast cancer cells. It also suggests that a combination of anti-cholesterol or lipid drugs, in combination with RTK inhibitors such as lapatinib, may benefit HER2+ breast cancer patients. Anti-cholesterol treatment paradigms offer new combinatorial strategies for HER2+ breast cancer that may be resistant to lapatinib or alternative anti-HER2 treatments.

### Metformin action against HER2+ breast cancer

We demonstrated that metformin has subtype specific effects on breast cancer cells, both *in vitro* and *in vivo*^[121-123]^. Metformin has been associated with increased survival of ER+/HER2+ breast cancer patients with metabolic dysregulation (type II diabetes) in several studies including the Phase III Adjuvant Lapatinib And/Or Trastuzumab Treatment Optimisation (ALTTO) trial^[124,125]^. The Phase II neoadjuvant metformin in combination with trastuzumab and chemotherapy in women with early HER2-positive breast cancer (METTEN) study studied neoadjuvant metformin, in combination with trastuzumab and paclitaxel, in early stage, HER2+ breast cancer and showed the combination was safe, with no significant toxicities attributable to metformin, and that metformin provided additional treatment benefit^[126]^. Other studies of metformin in HER2+ breast cancer patients are ongoing, as reviewed elsewhere^[127]^. Cufi *et al*.^[115]^ showed that the metformin-induced preferential killing of stem-like breast cancer cells was sufficient to overcome primary resistance to trastuzumab in HER2+ breast cancer *in vivo*. We also demonstrated that metformin is especially potent against trastuzumab resistant breast cancer cells via inhibition of HER2/IGF-1 receptor interactions^[123]^.

## Conclusion

HER2 targeting drugs have transformed both the clinical approach and prognosis of early- and late-stage HER2+ breast cancer. We describe many aspects of the complex biology of HER2-positive breast cancer, including cross-talk with myriad different pro-oncogenic signaling pathways, nuclear receptor, and steroid signaling, as well as lipid biosynthesis. Trastuzumab is an important and first-line inhibitor of HER2 signaling in breast cancer. Lapatinib with chemotherapy and alternate combinatorial agents, including RTK inhibitors, endocrine therapy, CDK 4/6 inhibitors, or cholesterol pathway inhibitors, holds promise for HER2+ breast cancer patients.

In the setting of primary or secondary resistance to lapatinib, a number of compensatory pathways may be activated including HER1, HER3, and HER4 or alternate plasma membrane receptors such as fibroblast growth factor receptor 1, MET, IGF-1R, or other tyrosine kinases [Table 2]. RTK signaling is also amenable to treatment by widely prescribed and low toxicity drugs including statins and metformin, based on their reliance on membranous lipid complexes. Metformin also kills breast cancer stem cells, inhibits proliferation and motility, and may induce apoptosis or autophagy.

**Table 2:** Mechanisms of resistance to lapatinib

Agent	Mechanism of resistance	Factors Involved	Target therapy to overcome resistance	Preclinical evidence
Lapatinib	HER2 signaling	HER2 mutation	Tesevatinib	[136]
		Heregulin-EGFR-HER3	Neratinib	[137]
	AXL Activation	PI3K/AKT/mTOR pathway alterations	Foretinib	[49]
		ER signaling pathway	Anti-estrogen therapies	[49]
	SRC Activatioln	PI3K/AKT/mTOR pathway alterations	Saracatinib/Dasatinib	[138]
			Saracatinib/Cetuximab	[139]
	Parallel/downstream pathways	PI3K/AKT/mTOR pathway alterations	NVP-BEZ235	[140]
		mTOR	AZD8055	[141]
		mTOR/p70S6K1 activation	Rapamycin	[142]
		mTOR	Rapamycin	[143]
	Immune system activation	PD-L1	Atezolizumab, Avelumab	[144,145]
		PD-L1	Pembrolizumab, Nivolumab	[144,146,147]
		CTLs	Lpilimumab, Tremelimumab	[101,144]
	Cell cycle regulation	Cyclin D1, CDK4/6 expression	Palbociclib/Ribociclib/abemaciclib	[84]
	ER signaling		Fulvestrant, Tamoxifen	[148]
			Aromatase inhibitors	[149]
	AR signaling		Enzalutaide	[99]
			Bicalutamide	[150]
			Enobosam	[151]

AR: androgen receptor; AXL: AXL receptor tyrosine kinase; CDK 4/6: cyclin-dependent kinase 4/6; CTLs: cytotoxic T-lymphocyte; EGFR: epidermal growth factor receptor; ER: estrogen receptor; HER2: human epidermal growth factor receptor 2; HER3: human epidermal growth factor receptor 3; IGF-1R: insulin-like growth factor 1 receptor; MAPK: mitogen activated protein kinase; MET: MET receptor tyrosine kinase; mTOR: mammalian target of rapamycin; PI3K: phosphoinositide 3-kinase; PgR: progesterone receptor; PD-1: programmed cell death-1; PD-L1: programmed cell death-1 ligand; Rb: retinoblastoma protein; RTKs: receptor tyrosine kinase; Src: SRC proto-oncogene non-receptor tyrosine kinase

As we discover additional mechanisms of lapatinib resistance, opportunities for new treatments will arise. We also need to develop more effective biomarkers to predict which combinations of agents will be most effective in overcoming resistance in individual breast cancer patients. Nonetheless, there have been many advances to overcome trastuzumab and/or lapatinib resistance in HER2+ breast cancer patients. Some of these, such as metformin or statins, are generally well tolerated in combination with targeted therapy and may provide additional benefits against other chronic diseases as well.

## Declarations

### Copyright

© The Author(s) 2020.
